# Teenage Rhinoplasty

**Published:** 2018-01

**Authors:** Abdoljalil Kalantar-Hormozi, Roozbeh Ravar, Ali Abbaszadeh-Kasbi, Nazanin Rita Davai

**Affiliations:** 1 Department of Plastic and Craniofacial Surgery, Medical College of Shahid Beheshti University of Medical Sciences, 15 Khordad Hospital, Tehran, Iran;; 2 School of Medicine, Tehran university of Medical Sciences, Tehran, Iran;; 3 Clinical Psychiatrist, Private Practice, Tehran, Iran

**Keywords:** Rhioplasty, Septorhinoplasty, Teenagers, Pediatric, Nasoseptal growth

## Abstract

**BACKGROUND:**

Rhinoplasty is among the most popular aesthetic surgical procedures selected by teenagers. When it comes to teenagers’ rhinoplasty, almost all surgeons believe that modified techniques should be considered because the nose is still growing. In this article, we prospectively followed teenagers who had undergone septorhinoplasty to assess the safety of procedure and its possible complications.

**METHODS:**

All the patients who were under 18 years old but for those who had a bleeding disorder, allergic rhinitis, and cleft lip nose were included in the study. All the patients were operated by the Senior author through closed rhinoplasty. Age, gender, indication for surgery, postoperative complications, need for revision surgery, postoperative satisfaction, and disturbance in facial growth until puberty were gathered for each of patients.

**RESULTS:**

Of all 40 patients, 38 (95%) patients were female and 2 (5%) patients were male. Mean age and follow up of patients was 16.1±0.8 years and 29.5±12.1 months, respectively. Fourteen (35%) patients had some degrees of nasal obstruction. Thirty-five (87.5%) patients expressed complete satisfaction with their rhinoplasty outcome. None of patients underwent revision rhinoplasty.

**CONCLUSION:**

The study indicates that patients’ craniofacial growth was not affected by the procedure, and it seems that septorhinoplasty is safe in teenagers.

## INTRODUCTION

According to the Cosmetic Surgery National Data Bank Statistics (2016), 39,709 candidates under the age of 18 underwent cosmetic surgery procedures. Within these surgical procedures, rhinoplasty, commonly known as a nose job, which is one of the most complex and challenging procedures of plastic surgery in which for aesthetic and functional goals surgeon reshape the components of the nose is a popular surgical procedure among teenagers.^1^^,^^2^

Rhinoplasty in teenagers is more crucial than in adults because craniofacial is still growing in teenagers, unlike adults, and altering cartilaginous structure may lead to complications, postsurgical distortion, or disturbance of craniofacial growth. Moreover, secondary rhinoplasty rate is greater in teenagers than in adults. On the other hand, chronic rhinitis, turbinate and adenoid hypertrophy, choanal atresia, and deviated nasal septum leading to nasal obstruction may lead to growth inhibition of the nose, paranasal sinuses, and midface, so performing septorhinoplasty is helpful when indicated.^3^^-^^11^

Even though studies indicating safety of rhinoplasty in teenagers are increasing, patients and their families should be counseled regarding possible complications, possibility of secondary rhinoplasty, and even its possible adverse effects on craniofacial growth, and also, psychologic counselling should be considered for whom aesthetic aspects is initial motivation.^12^^-^^16^ Here, we conducted a prospective study involving teenage patients who were the candidates for septorhinoplasty to assess the safety of procedure among them and possible complications. 

## MATERIAL AND METHODS

This is a prospective study carried out in a private setting from January 2013 to April 2017. All the patients who were under 18 years old and underwent septorhinoplasty were included in the study. Patients with a bleeding disorder, allergic rhinitis, or cleft lip nose were excluded from the study.^3^ A throughout medical history and physical examination were obtained for all the patients. Routine lab tests as well as radiographic imaging were performed for all the patients. Further tests were tailored to each individual conditions. All the patients were operated by the Senior Author, and closed technique for rhinoplasty was applied for all the patients. Age, gender, indication for surgery, postoperative complications, need for revision surgery, postoperative satisfaction, and disturbance in facial growth until puberty were gathered for each of patients. 

**Fig. 1:**
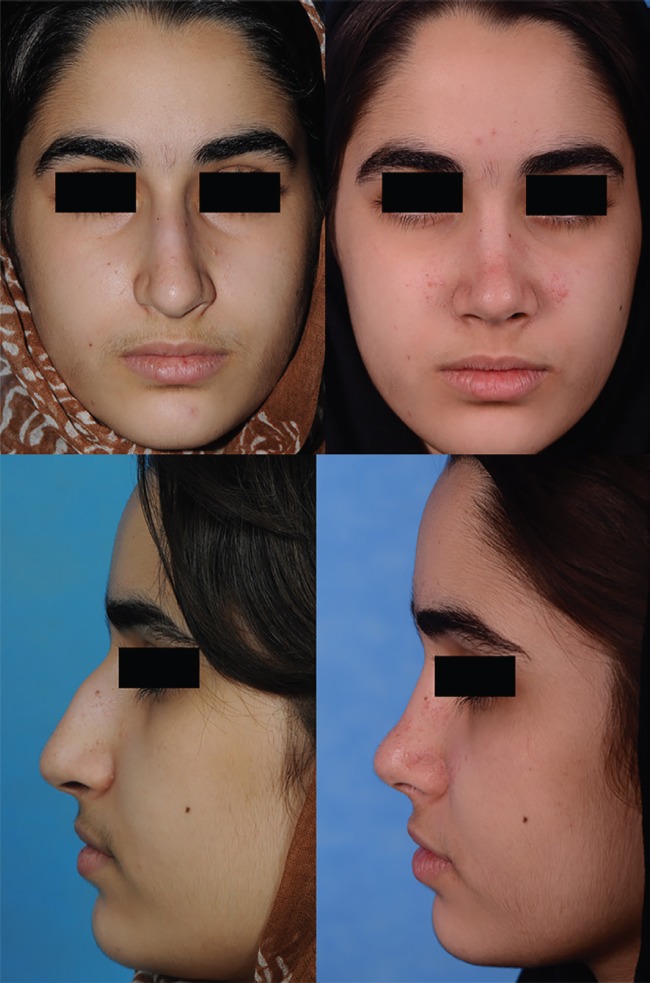
A 14-year old girl who underwent rhinoplasty. Left sided photos were taken before the rhinoplasty (Preoperative photos). Right sided photos were taken after a 60-month period (Postoperative photos).

The youngest patient was a 14-year-old girl whose bone age study had demonstrated structural maturity (Figure 1) before the septorhinoplasty. Statistical analysis was accomplished using the Statistical Package for Social Sciences (SPSS 16, SPSS Inc., Chicago, US). Data are expressed as number (%) or mean±standard deviation (SD).

## Results

**Fig. 2:**
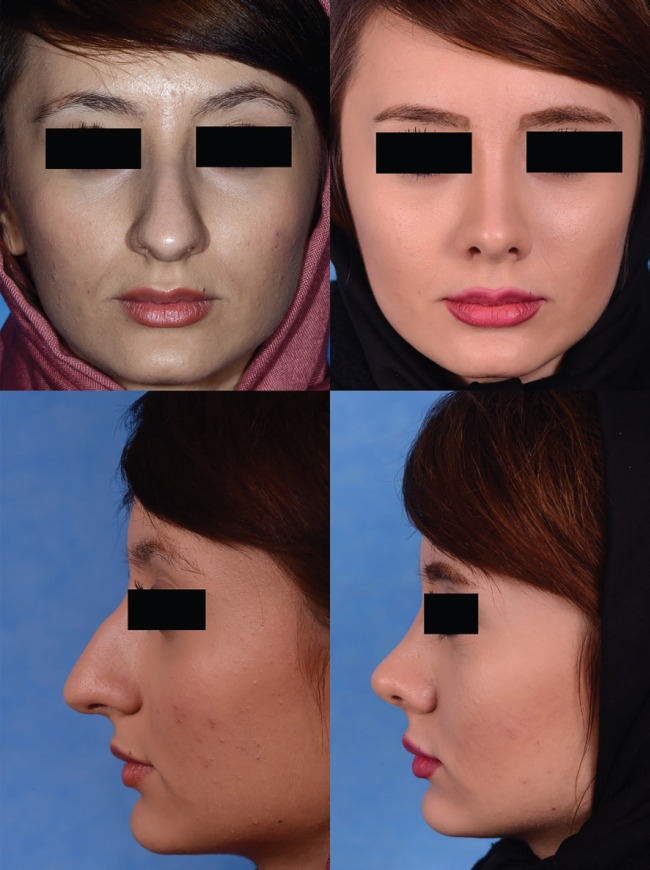
A 17-year old girl who underwent rhinoplasty. Left sided photos were taken before the rhinoplasty (Preoperative photos). Right sided photos were taken after a 12-month period (Postoperative photos).

Forty patients were included in this study, and 38 (95%) patients were female while 2 (5%) of those were male. Mean age of patients was 16.1±0.8 years (Table 1). Indications for surgery for 40 (100%) patients were aesthetic, and, also, 14 (35%) of these patients had some degrees of nasal obstruction. Among these 14 patients, Cottle test was positive but external nasal valve collapse was negative. Mean follow up period was 29.5±12.1 months for all the patients. Thirty-five (87.5%) patients expressed complete satisfaction with their rhinoplasty outcome. None of patients underwent revision rhinoplasty (Figure 2). Table 2 outlines postoperative complications (both short term and long term). Craniofacial growth of patients was not affected by the procedure in any patients.

**Table 1:** Characteristics of patients.

**Variable**	**Male Patients**	**Female Patients**	**Whole Patients**
Number N (%)	2 (5)	38 (95)	40 (100)
Age Mean±SD	17.1±0.7	16.1±0.7	16.1±0.8
Follow up period Mean±SD	12.5±0.7	26.3±8.2	29.5±12.1
Primary motivation			
Only Aesthetic N (%) Only Functional N (%) Both N (%)	0 (0.0) 0 (0.0) 2 (14.2)	26 (100) 0 (0.0) 12 (85.8)	26 (100) 0 (0.0) 14 (100)
Revision Surgery N (%)	0 (0.0)	0 (0.0)	0 (0.0)

**Table 2:** outlines both short term and long term complications of surgery.

**Variable**	**Number of patients No (%)**
Septal hematoma	0 (0.0)
Infection	1 (2.5)
Nasal deviation	0 (0.0)
Tip depression	0 (0.0)
Wide dorsum	0 (0.0)
Short nose	0 (0.0)
Nostril asymmetry	0 (0.0)
Broad nasal bones	0 (0.0)
Transient nasal pain	0 (0.0)
Worsening nasal obstruction	0 (0.0)
Swelling around stitches	2 ( 5)
Epistaxis	1 ( 2.5)
Graft migration or resorption	0 (0.0)
Disturbing facial growth	0 (0.0)
Satisfaction with the outcome	
Low Deep	5 (12.5) 35 (87.5)

## DISCUSSION

Anatomically speaking, in comparison to adults, teenagers have a greater nasal cartilage-to-bone ratio and a larger nasolabial angle as well as less projected dorsum and nasal tip.^4^^,^^17^ Meanwhile, the nose continues to grow until some 12 to 16 years of age in girls and 15 to 18 years of age in boys, so any acquired or congenital nasal abnormalities leading to abnormal nasal growth must be corrected through elective rhinoplasties, such as septoplasty, rhinoplasty, rhinosep-toplasty, to restore normal nasal growth, function, and aesthetics.^11^^,^^18^^-^^22^

To the best of our knowledge, Freer and Killian, in 1902 and 1905, respectively, were among the first surgeons who performed septorhinoplasty in pediatrics, and consequently the procedures including wide resection of cartilages led to the severe disturbance of nasal growth and retropositioning of the maxillary bone.^23,24^ Yet, today, with the application of more conservative approaches septorhinoplasty in teenagers is performed without any major consequences for the craniofacial growth.^4^

Besides closed septorhinoplasty technique, open septorhinoplasty, external septorhinoplasty, may be used for teenagers’ septorhinoplasty. Although external septorhinoplasty, indicated to operate dermoid cyst, septal abscess, cleft lip nose, or severe septal deformity, has more advantages than closed septoplasty, including better access to the nasal septum to manipulate the nasal components, there was a tendency for the operated noses through this method to be shorten after completing craniofacial growth as well as columellar scar.^25^^-^^29^

Although, based on several studies, open septoplasty should be postponed until after the age of 16 years, patients suffering from severe septal deformities causing nasal obstruction and consequently adverse effects on craniofacial growth should undergo septoplasty regardless of age, even at birth.^5^^,^^7^^,^^14^^,^^30^^-^^33^ Among operated patients in the study, we did not have any long term problems, but 4 (10%) patients had short term complications, not severe, resolved without any sequelae within a few hours to days. Also, 35 (87.5%) patients had deep satisfaction with the outcome of the rhinoplasty. 

Crysdale and Tatham reported that approximately 70% of their patients had satisfactory outcome.^25^ Koltai *et al* mentioned neither postop complications nor long term complications.^34^ Locke and Kubba in 2011 reported that their rhinoplasties did not affect the craniofacial growth and all the patients were satisfied with their postoperative nasal appearance.^35^ Chung *et al* in 2014 reported that all of their patients after a 90 day period were satisfied with the outcome of their operation.^36^ Constantian in 2012 reported that 97% of his patients expressed happiness with their postoperative outcome.^9^

Within the operated patients, there was no need for the revision surgery, and Constantian explicated that the major reason why patients undergo revision surgery is development a new deformity after the primary rhinoplasty.^9^ In a study by Neaman *et al* in 2013, including both adults and teenagers, they reported that the revision surgery rate was 9.8%.^37^ Moreover, it is claimed that revision surgery rate in teenagers is greater than that of adults.^37,38 ^Male to female ratio was 0.05 in current study, but some studies suggest that male to female ratio is greater than that of our study because boys are more prone to engage in high contact sports or street fighting.^39^^-^^41^ Current study revealed that craniofacial growth was not affected by the procedure among teenagers who had undergone closed septorhinoplasty, and, hence, septorhinoplasty may be safely performed when is indicated. 
